# Ureter Injury in Laparoscopic Para-Aortic Lymphadenectomy for Endometrial Cancer by the Transperitoneal Approach

**DOI:** 10.1155/2023/3138683

**Published:** 2023-09-19

**Authors:** Hiroharu Kobayashi, Misa Kobayashi, Yoshihiro Takaki, Yuki Kondo, Yuri Hamada, Haruhiko Shimizu, Yumi Shimizu, Masaru Nagashima, Hiroshi Adachi

**Affiliations:** Department of Gynecology, Seirei Hamamatsu General Hospital, Japan

## Abstract

The patient was 66 years old, had three pregnancies and two deliveries, and was menopausal at the age of 51. She had irregular bleeding and was found to have a chicken-egg-sized uterus and a thickened endometrium (23 mm). She underwent laparoscopic surgery for uterine endometrial cancer (endometrioid carcinoma G1, stage IB). Laparoscopic simple hysterectomy, bilateral adnexectomy, pelvic lymph node dissection, para-aortic lymph node dissection, and partial omentectomy were performed using the transperitoneal approach (TPA). The patient was obese, with a height of 148 cm, a weight of 68 kg, and a body mass index of 31 kg/m^2^. She had a large amount of visceral fat, which made it difficult to expand the surgical field during para-aortic lymph node dissection. A laparoscopic fan retractor (EndoRetract II, Medtronic) was used to lift the intestinal tracts and expand the field of view. It broke the fat around the left kidney, and the exposed left ureter was heat-damaged using a vessel sealing device (LigaSure, Medtronic). Postoperatively, a left ureteral stent was placed, and continuous urine draining into the retroperitoneum was performed. To prevent injury to the left ureter, the left ovarian vein branching from the left renal vein should be exposed as a landmark before the left ureter running parallel to it is isolated. It is essential that the fat around the left kidney is not broken during this operation. The left iliopsoas muscle should be exposed, and using this as a base, the left ovarian vein, left ureter, and left perirenal fat should be compressed and moved to the left side using a fan retractor to ensure a safe operation.

## 1. Introduction

Laparoscopic para-aortic lymphadenectomy for uterine endometrial cancer can be performed using the transperitoneal approach (TPA) or the extraperitoneal approach (EPA), and there are several reports on the safety of these approaches [1–7]. In EPA, the extraperitoneal cavity is expanded to allow the complete elimination of the intestinal tracts from the field of view. TPA has the advantage of being able to remove the uterus first, which allows rapid pathological examination with intraoperative frozen sections, but the problem is how to exclude the intestinal tracts from the field of view. We report a case in which the left ureter was injured during para-aortic dissection by TPA. The large amount of visceral fat made it difficult to expand the field of view.

## 2. Case Presentation

The patient was 66 years old, had 3 pregnancies and 2 deliveries, and was menopausal at age 51. The patient's medical history included breast cancer and appendicitis. She had irregular bleeding and was found to have a chicken-egg-sized uterus and a thickened endometrium (23 mm). MRI showed a 37 × 40 × 32 mm mass in the uterine lumen with the possibility of slightly more than 1/2 myometrial invasion. Computed tomography (CT) revealed no distant metastasis or enlarged lymph nodes. The preoperative diagnosis was uterine endometrial cancer, endometrioid carcinoma G1, stage IB (FIGO2008). Laparoscopic surgery was then performed using the transperitoneal approach (TPA). First, the uterus and bilateral adnexa were removed, and rapid intraoperative pathology revealed endometrioid carcinoma G2 with more than 1/2 myometrial invasion. The patient underwent pelvic lymph node dissection, para-aortic lymph node dissection, and partial omentectomy. The patient was obese (height, 148 cm; weight, 68 kg; body mass index, 31 kg/m2) and had abundant adipose tissue around the intestinal tracts, which made it difficult to expand the field of view during para-aortic dissection. The surgeon was unaware of any obvious intraoperative injury to the ureter, but an intraoperative cystoscopy performed at the end of the surgery failed to confirm urine outflow from the left ureteral orifice. The operative time was 8 h and 45 min, and the blood loss was 125 mL. On postoperative day 1, the patient had left back pain and elevated serum creatinine (1.36 mg/dL), and retrograde urography showed leakage of contrast medium from the upper ureter, suggesting that the left ureter was damaged. A left ureteral stent was then inserted. On postoperative day 9, computed tomography (CT) showed encapsulated fluid accumulation in the left retroperitoneal space, which was considered leaking urine retention, and continuous puncture drainage was performed. Two months after surgery, the retroperitoneal fluid accumulation disappeared on CT, and the drainage tube was removed. Ten months after surgery, the left ureteral stent was removed. The final pathology was endometrial cancer, endometrioid carcinoma G2, myometrial invasion 16 mm/18 mm, vascular invasion+, two lymph node metastases+ (left external iliac lymph node and lymph node between the aorta and inferior vena cava), no metastasis to the omentum, pT1bN2M0, and stage IIIC2 (FIGO2008). The patient underwent adjuvant radiation therapy. She was alive and disease-free at 1 year and 6 months after surgery.

**Figure 1:**
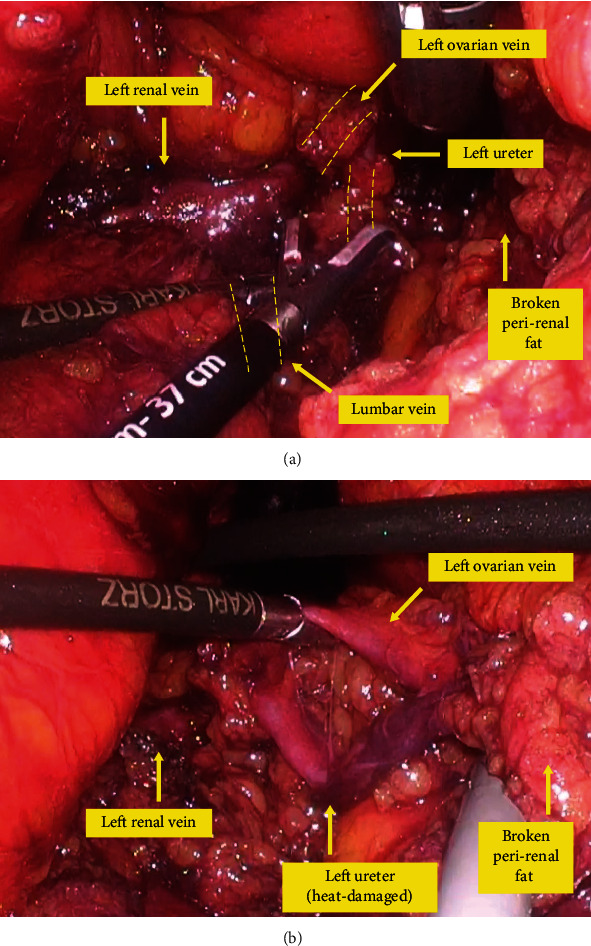
Still images of the intraoperative video (the case of left ureter injury). (a) The left ureter within the fat, along with the left ovarian vein, was trapped by the fan retractor. (b) The trapped left end of the left ureter was thermally damaged by the sealing device.

A review of the operative video showed that during dissection just below the left renal vein, a laparoscopic fan retractor (EndoRetract II, Medtronic) bit into the perirenal fat and crumbled it, and the exposed left ureter was thermally damaged using a vessel sealing device (LigaSure, Medtronic). It could be seen from the image that the left ureter within the fat, along with the left ovarian vein, was trapped by the fan retractor (Figure 1(a)). This trapped left end was thought to have been thermally damaged by the sealing device (Figure 1(b)).

Written informed consent for the surgical video to be used anonymously for clinical research was obtained preoperatively from the patient.

## 3. Discussion

**Figure 2:**
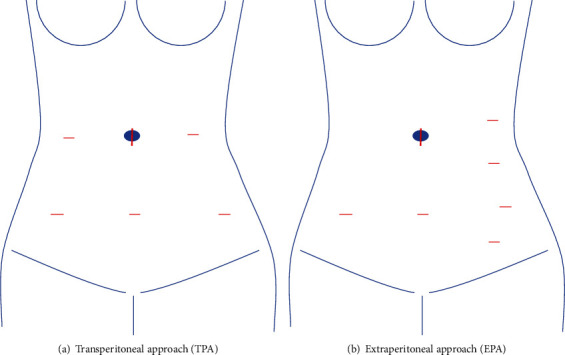
Port placement of the trocars for laparoscopic para-aortic lymphadenectomy in our hospital.

The trocar position of laparoscopic surgery for uterine endometrial cancer using TPA in our hospital is shown in Figure 2(a). The camera is inserted through the navel, and three ports were created in the lower abdomen. The surgeon stands on the patient's left side and uses the midline lower abdomen and left lower abdomen ports. The patient is positioned in a 20-30° head-down position. The uterus and bilateral adnexa are first removed, and the histology and degree of myometrial invasion are evaluated by rapid pathology using intraoperative frozen sections. Usually, if the histology is high-grade, such as endometrioid carcinoma G3 or serous carcinoma, or if there is more than half myometrial invasion, para-aortic lymph node dissection is performed. After pelvic lymph node dissection, a camera is inserted through the midline of the lower abdomen, and the surgeon stands to the right of the patient to perform para-aortic lymph node dissection and partial omentectomy using two ports: the right lateral abdomen and right lower abdomen, or the right lateral abdomen and umbilicus. The incised peritoneum is pulled at several places with a needle thread and suspended on the abdominal wall using Lapa-Her-Closure (Hakko). The intestines are compressed and moved using a laparoscopic fan retractor (EndoRetract II, Medtronic) to develop a field of view. For reference, the trocar position of EPA with the left lateral abdominal approach in our hospital is shown in Figure 2(b).

**Figure 3:**
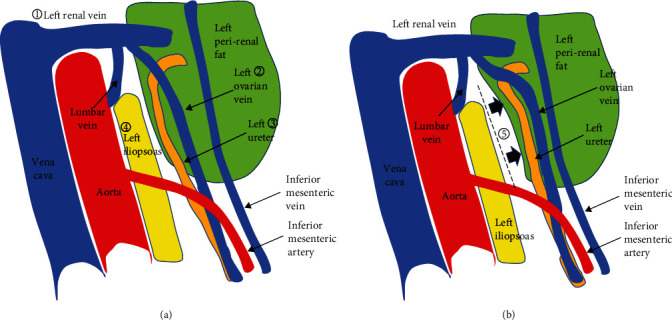
(a) Left ovarian vein and left ureter running on the surface of the perirenal fat. (b) Left perirenal fat with left ovarian vein and left ureter is compressed and moved to the left side using the left iliopsoas as a base point.

**Figure 4:**
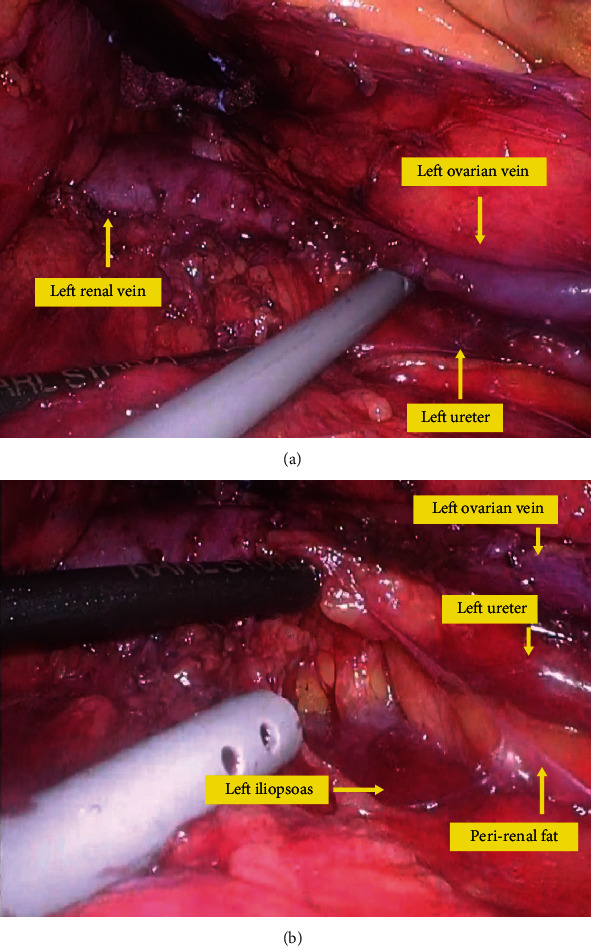
Still images of the intraoperative video (the case of safe surgical procedure). (a) After the surgical field developed to the level of the left renal vein, the left renal vein and left ovarian vein flowing into it were exposed. (b) The left ureter, which ran parallel to the left ovarian vein, was identified by expanding the space in the dorsal direction of the left ovarian vein without breaking the left perirenal fat.

This patient had a large amount of visceral fat. Moreover, when the intestinal tracts were compressed and moved with the fan retractor, the fat around the left kidney was broken, and the exposed left ureter was thermally damaged by the vessel-sealing device. In the para-aortic region under the left renal vein, the left ureter runs parallel to the left ovarian vein on the surface of the left perirenal fat. Close to the left renal vein, the left ureter changes direction and heads toward the left kidney, and the left ovarian vein flows into the left renal vein (Figure 3(a) ①, ②, and ③). This region is covered with thin, overlapping layers of mesentery and Gerota's fascia, and it is difficult to identify these membranes individually. This is probably the most difficult part of expanding the surgical field during para-aortic lymph node dissection. In this case, the left renal vein was exposed, but the left ovarian vein branching from it was not exposed. If the left ovarian vein had been exposed as a landmark, injury to the left ureter could have been prevented.  Figure 4 shows an image of another case of TPA for para-aortic dissection in our hospital. In this case, there was less fat, and the surgical field was easier to expand than in the present case. After the surgical field developed to the level of the left renal vein, the left renal vein and left ovarian vein flowing into it were exposed (Figure 4(a)). The left ureter, which ran parallel to the left ovarian vein, was identified by expanding the space in the dorsal direction of the left ovarian vein (Figure 4(b)). The left ureter and left ovarian vein ran along the surface of the left perirenal fat, which was not broken with careful manipulation in this case. Next, the left iliopsoas muscle was exposed (Figure 3(a) ④), and using it as a base, the left perirenal fat with the left ovarian vein and left ureter was compressed and moved to the left side with a fan retractor (Figure 3(b) ⑤). It is important not to break the left perirenal fat, as this will result in a very poor visual field in laparoscopic para-aortic lymphadenectomy by TPA.

The merit of EPA is that the intestinal tracts can be completely removed from view, which can be especially advantageous in the surgery of obese patients. A study showed that patients with a higher BMI had lower aortic node yields by TPA but not EPA [8]. The disadvantage of EPA is that rapid pathology of the uterus cannot be performed prior to para-aortic lymph node dissection. Moreover, in the left-sided abdominal approach of EPA (Figure 2(a)), lymph node dissection between the aorta and inferior vena cava or on the right side of the inferior vena cava becomes somewhat difficult due to the field of view. Peng et al. reported EPA with an umbilical single port, which allows EPA to have a view from the caudal side, similar to TPA [9]. For TPA, Mizumoto et al. reported an approach from the left side of the descending colon called left dome formation, in which the left ovarian vein and left ureter can be followed all the way from the caudal side, ultimately providing a view exactly like that of the left-sided approach of EPA [10]. In the approach from the right side of the descending colon of TPA (our case), it is difficult to follow the left ovarian vein from the caudal side to the left renal vein, and it would be better to follow it in the opposite direction from the left renal vein to the caudal side. It is important to avoid destroying the left perirenal fat when exposing the left ureter, which runs parallel to the left ovarian vein. Furthermore, using the exposed left iliopsoas as a base, the left ovarian vein, left ureter, and left perirenal fat should be compressed and moved to the left direction by a fan retractor, which would prevent left ureter injury.

One method that could have been used to avoid ureteral injury in this case was ureteral stenting. Ureteral stents provide tension to the ureter and make the ureter very easy to identify. If the ureter is injured, it increases the likelihood that the injury can be detected and repaired intraoperatively. The drawback is that it takes time to insert and remove the ureteral stent. Preoperative ureteral stenting should have been considered in this case with obesity. Once the perirenal fat is broken, it is difficult to continue the laparoscopic procedure. One way to address this would be to construct a new trocar port, increase the number of assistant forceps, and gently eliminate the intestinal tract to the extent possible with a fan retractor. If it is difficult to secure the surgical field even with these methods, we have to abandon the lymph node dissection just below the left renal vein or convert to open surgery.

## Data Availability

The data used to support the findings of this study (still images of the intraoperative videos) are included within the article.
